# Acute Pancreatitis: Current Clinical Approaches, Molecular Pathophysiology, and Potential Therapeutics

**DOI:** 10.1097/MPA.0000000000002259

**Published:** 2023-06-12

**Authors:** Mark B. Wiley, Kunaal Mehrotra, Jessica Bauer, Cemal Yazici, Agnieszka B. Bialkowska, Barbara Jung

**Affiliations:** From the ∗ Department of Medicine, University of Washington, Seattle, WA; † Department of Medicine, University of Illinois Chicago, Chicago, IL; ‡ Department of Medicine, Renaissance School of Medicine at Stony Brook University, Stony Brook, NY.

**Keywords:** acute pancreatitis, SIRS, cytokines, Activin A

## Abstract

**Objective:**

Severe acute pancreatitis (SAP), pancreatic inflammation leading to multiorgan failure, is associated with high morbidity and mortality. There is a critical need to identify novel therapeutic strategies to improve clinical outcomes for SAP patients.

**Materials and Methods:**

A comprehensive literature review was performed to identify current clinical strategies, known molecular pathophysiology, and potential therapeutic targets for SAP.

**Results:**

Current clinical approaches focus on determining which patients will likely develop SAP. However, therapeutic options are limited to supportive care and fluid resuscitation. The application of a novel 5-cytokine panel accurately predicting disease outcomes in SAP suggests that molecular approaches will improve impact of future clinical trials in AP.

**Conclusions:**

Inflammatory outcomes in acute pancreatitis are driven by several unique molecular signals, which compound to promote both local and systemic inflammation. The identification of master cytokine regulators is critical to developing therapeutics, which reduce inflammation through several mechanisms.

Acute pancreatitis (AP) related healthcare expenses exceed $2.5 billion per year, and the number of emergency department visits for AP has increased by 18% over the last 15 years.^1,2^ Despite climbing incidence, research on pancreatitis has decreased more than any other GI disease over 50 years. In addition, the number of clinical studies is limited with significant issues surrounding patient recruitment.^3–9^ This has led to a considerable gap in knowledge of disease etiology and treatment options.

It was proposed over 115 years ago that AP is caused by premature activation of trypsinogen to trypsin in the pancreas, stimulating pancreatic injury and subsequent inflammation.^10^ Premature trypsinogen activation has been confirmed in several animal models, further supporting this hypothesis.^11–14^ Despite the role of trypsin digestion of tissue in the initiation of AP, clinical trials with trypsin inhibitors have shown no benefit for patients.^15^

Approximately 80% of AP cases are related to alcohol abuse or gallstone disease.^16^ The remainder of cases occur post–endoscopic retrograde cholangiopancreatography or are classified as idiopathic.^17^ Social and cultural risk factors also alter AP incidence as for instance increases in ethanol-related AP are observed in the United Kingdom during the Christmas and New Year weeks.^17^ Several other risk factors that contribute to progression of mild AP to SAP include the following: sex, age, diet, and body mass index; however, they do not seem to cause disease.^17^ Nicotine has been shown to impair pancreatic blood flow and increase pancreatic acinar cell Ca^2+^ levels suggesting that cigarette smoke can directly induce pancreatitis.^18–20^

Many of the early events observed in AP occur in acinar cells, where disruptions in Ca^2+^ signaling are observed leading to local and systemic inflammation.^21–23^ The acute inflammatory response during AP is followed by a cytokine surge and systemic inflammatory response syndrome (SIRS), which is associated with early organ injury.^24–27^ There is a second phase of AP, which includes a systemic anti-inflammatory response. This phase is associated with a significant risk of infection in the pancreatic fluid caused by translocation of intestinal microbiota due to the failure of the intestinal barrier.^28^ This results in 2 peaks of mortality in the early phase due to organ failure and in the late phase due to infection.^28^ Acute pancreatitis severity is classified into mild, moderate, or severe disease based on the Revised Atlanta Classification.^24^ Those with SAP have mortality rates up to 30%, with nearly half of those deaths occurring within 14 days of diagnosis, driving the need for early therapeutic markers and intervention methods.^29,30^

## CURRENT CLINICAL STRATEGIES

Currently, there are no validated models to predict AP severity early on, and therapeutic options are limited to supportive care.^29,31–34^ However, several diagnostic tools exist that attempt to classify patients into subgroups to determine whether admission into the intensive care unit (ICU) is probable.

### Diagnostic Tools

Contrast-enhanced computed tomography is the preferred imaging technique for determining the extent of pancreatic inflammation and identifying complications such as (peri)pancreatic necrosis stemming from AP.^31,34^ However, the risk associated with repeated radiation exposure limits contrast-enhanced computed tomography as a monitoring tool for AP patients. Magnetic resonance imaging is preferred when minimal radioactive exposure or better evaluation of the pancreatic duct is required.^31,35^ Although imaging evidence of pancreatitis is only one of the three criteria for diagnosis of AP, these modalities are also helpful in identifying potential complications. Right upper quadrant ultrasound is also used to identify gallstones as a potential AP etiology.^33–37^ Endoscopic ultrasound can be performed to screen for microlithiasis or choledocholithiasis when clinical suspicion is high, but right upper quadrant ultrasound is negative.^34^ Disease severity is determined using either the Revised Atlanta Classification or the Determinant-Based Classification of Acute Pancreatitis Severity.^24,38^ Each of these classification systems was published in the same year, with similar categories for patients based on organ failure. Both classification systems have been compared extensively across several studies, with no significant difference being observed between the two in their ability to accurately classify the severity of AP in subgroups of patients.^39–41^

### Prognostic Tools

The Ranson score was among the first scoring systems aiming to classify the severity of AP. It was designed primarily for alcohol-induced AP, but the minimum criteria were modified to assess gallstone pancreatitis.^42^ The Glasgow score includes key clinical and biochemical variables (Table 1) and is considered a useful prognostic tool for mortality regardless of etiology.^43^ The primary disadvantage to both of these scoring systems includes the 48-hour requirement for calculation.

**TABLE 1:** Criteria Used for Each Prognostic Tool Available to Classify and Predict Disease Severity in AP

Variable	Ranson^42^	Glasgow^43^	APACHE-II^44^	BISAP^45^	PASS^46^
Age	>55 y/o	>55 y/o	+	>60 y/o	−
WBC	>16,000 cells/μL	>15,000 cells/μL	+	(See SIRS)	(See SIRS)
Glucose	>200 mg/dL	10 mmol/L	−	−	−
AST	>250 IU/L	−	−	−	−
ALT	−	−	−	−	−
LDH	>350 IU/L	>600 IU/L	−	−	−
Calcium	<8.0 mg/dL	<2.0 mmol/L	−	−	−
Hematocrit	Decrease by 10%	−	+	−	−
Blood pH	Base deficit >4 mEq/L	−	+	−	−
Sequestration of fluids	>6 L	−	−	−	Part of organ failure
Albumin	−	<32 g/L	−	−	−
Arterial po_2_	<60 mm Hg	<60 mm Hg	+	−	Part of organ failure
BUN	Increase by 5 mg/dL	>16 mmol/L	−	>25 mg/dL	Part of organ failure
Mental status	−	−	+	+	−
Pleural effusions	−	−	−	+	−
Body temperature	−	−	+	−	(See SIRS)
Sodium	−	−	+	−	−
Potassium	−	−	+	−	−
Creatinine	−	−	+	−	Part of organ failure
Organ failure	−	−	+	−	+
Blood pressure	−	−	+	−	Part of organ failure
Abdominal pain	−	−	−	−	1–10
Tolerance of solid food	−	−	−	−	+
Morphine equivalent dose	−	−	−	−	+
SIRS	−	−	+	HR >90 bpm, RR >20 breaths/min or pco_2_ < 32 mm Hg, and WBC <4000 or >12,000 cells/μL	HR >90 bpm, RR >20 breaths/min, body temp <36°C or >38°C, and WBC <4000 or >12,000 cells/μL
Score outcome	0–2 = 0%–3% mortality 3–4 = 15% mortality 5–6 = 40% mortality 7–11 ≈ 100% mortality	≥3 = severe AP	>8 = severe AP	Aged group ≥3 severe AP probable, younger group ≥2 severe AP probable	>140 = moderately severe and severe AP

The APACHE-II and PASS scoring systems both have a standard range of values for each variable to determine how many points the variable contributes to the final score. All five of these prognostic tools provide a robust method for determining disease severity in AP patients.

AST, aspartate aminotransferase; ALT, alanine aminotransferase; BUN, serum urea nitrogen; HR, heart rate; LDH, lactate dehydrogenase; RR, respiratory rate; SIRS, systemic inflammatory response syndrome; WBC, white blood cells.^42–46^

The Acute Physiology and Chronic Health Evaluation II (APACHE-II) score was intended to evaluate patients with acute illness admitted to the ICU and is an accurate predictor of mortality in AP.^31,44,47,48^ The Bedside Index of Severity in Acute Pancreatitis (BISAP) score was developed in 2008 and can be calculated within 24 hours of admission.^45^ The BISAP system relies upon the Classification and Regression Tree analysis and continues to be credited as an accurate and valid method for predicting patient outcomes in AP.^49–51^ The Pancreatitis Activity Scoring System (PASS) includes the following 5 parameters: SIRS, abdominal pain, opiate requirement, organ failure, and oral intake tolerance.^46^ Recently, the PASS was updated to remove the morphine equivalent dosage (opiate requirement) as a parameter to improve SAP prediction.^52^ A summary of these 5 prognostic tools is included in Table 1.

### Therapeutic Options

Currently, therapeutic options include adequate resuscitation, especially during initial presentation, early feeding, and maximizing supportive care if patients develop local/systemic complications. Clinical findings suggest that early fluid resuscitation is critical to improving outcomes in AP.^53^ Lactate has been shown to reduce IL-1β production both in vitro and in vivo,^54^ which may account for the evidence suggesting that patients receiving lactated Ringer's solution instead of saline have reduced ICU admission rates and reduced length of hospital stay.^55^ Recent data obtained from the WATERFALL clinical trial suggests that aggressive (20 mL/kg bolus for 2 hours, then 3 mL/kg/hour infusion) fluid resuscitation via lactated Ringer's solution increases the risk of volume overload without improvement in primary outcome of AP patients when compared with moderate (10 mL/kg bolus only if hypovolemic, then 1.5 mL/kg/hour infusion) fluid resuscitation.^56^ This data is supported by evidence that aggressive hydration is associated with worse outcomes in patients with systemic inflammatory diseases including sepsis,^57^ acute lung injury,^58^ and critical care patients.^59^ The American Gastroenterology Association guidelines strongly suggest early (within 24 hours) oral feeding when tolerated.^32^ Antibiotics are recommended only if an infection has been confirmed.^31,60–62^ No effective pharmacological treatments have been identified despite the completion of several clinical trials.^31,63–66^ Promising preliminary data from a recently completed study suggest that targeting Ca^2+^ signaling in AP patients via the Orai1 inhibitor (Auxora) may improve outcomes; however, more human trials are necessary to determine the safety and efficacy of this therapeutic.^67^

Furthermore, existing health inequalities in AP will likely limit the availability of potential therapeutic agents in minorities. African Americans are at an increased risk for AP and have higher rates of organ failure and mortality.^68–70^ Similar disparities are observed in other populations, as challenges with timely access to care during AP attacks are also seen in Hispanics.^71^ Therefore, reporting race and ethnicity data in clinical AP studies are crucial for potential subgroup analysis and can increase our ability to address health inequities in AP.^72^

## THE PROINFLAMMATORY RESPONSE TO AP

Several cytokines have been found to be upregulated in SAP patients, which are likely to contribute to inflammatory responses locally and systemically. Severity of AP is positively correlated with the intensity of inflammation, indicating that the development of therapeutics targeting inflammatory cytokines may inhibit disease progression.^73–75^

### Inflammatory Cytokines of AP

Several inflammatory cytokines are upregulated in circulation in patients with SAP.^31,47^ Key molecules regulating cytokine cascade must be identified to aid in development of life-saving therapeutics in SAP patients.

#### Acute Phase Response in AP

The acute phase response is a broad term used to describe the reaction in an organism shortly after an insult.^76^ This response includes several proteins upregulated or downregulated in response to the proinflammatory cytokines: tumor necrosis factor alpha (TNF-α), interleukin-1β (IL-1β), and IL-6.^76–78^ Pancreatic acinar cells produce TNF-α and its receptor and, through this signaling, mediate injury-induced cell death.^79^ However, previous research identified no differences in the levels of TNF-α in serum between patients who developed severe or mild AP. In addition, polymorphisms in the gene encoding TNF-α are not associated with an increase in AP susceptibility.^80,81^ Interleukin-1β levels have been shown to correlate with severity of AP; however, mechanistic studies of how IL-1β signaling may be driving disease are lacking.^82,83^ The primary induction of acute phase protein production is caused by IL-6, produced by macrophages.^47,76,77^ In a cerulein-induced mouse model of AP, IL-6 knockout mice displayed an increased survival rate, which was reversed when exogenous IL-6 was administered.^84^ In addition, this study proved that IL-6 signals through STAT3 in acinar cells to stimulate CXCL1 production, a neutrophil chemoattractant, to drive disease progression.^84^ These findings were recently further confirmed in a study that found that disease progression and inflammatory cytokine production are reduced via manipulation of upstream regulators of the IL-6/STAT3 signaling axis in vitro and in vivo.^85^ The early release of IL-6 provides an attractive marker of progression in AP; however, rapid IL-6 clearance and the cost of IL-6 quantitation provide major drawbacks for widespread use in the clinic.^86–89^

C-reactive protein (CRP) is an acute phase reactant produced in the liver in response to IL-6 and has been used in diagnosis, prognosis, and mortality prediction in patients with severe inflammation.^78^ Traditionally, CRP has been used in the clinic to monitor AP progression, where it has been reported to increase in circulation with disease progression.^90–92^ However, conflicting evidence indicates that the timing of CRP measurement and quantitation of other serum markers (ie, IL-6) are critical to predicting disease outcomes.^93^ Furthermore, a recent retrospective analysis determined that CRP measurement at admission or at 48 hours does not predict the likelihood of developing complications from AP.^93^ In addition, liver disease may alter CRP production, which may be a significant factor in patients experiencing AP stemming from alcohol abuse.^94,95^

Procalcitonin has been identified to be a key marker in circulation for patients experiencing severe bacterial infections and/or multiorgan failure.^96^ Measurement of this molecule has been shown to predict the risk for development of severe AP and infected pancreatic necrosis with a sensitivity of 0.72 and 0.80, respectively, and a specificity of 0.86 and 0.91, respectively.^97^ Synthesis of physiologic blood proteins (albumin, transferrin, and others) is downregulated in the acute phase response, and lower levels of circulating albumin throughout AP are predictive of organ failure and mortality.^76,98,99^ Despite these published studies surrounding the acute phase proteins and their clinical relevance, very few mechanistic studies have been published implicating these proteins, limiting the advancement of AP clinical trials.

#### Damage-Associated Molecular Patterns and the Complement Cascade in AP

Early in the disease, an inflammatory injury will lead to leakage of prematurely activated pancreatic enzymes, further stimulating apoptosis, autophagy, necrosis, and release of damage-associated molecular patterns (DAMPs).^100,101^ These DAMPs recruit innate immune effector cells, generating an inflammatory response in the tissue.^31,100^ Paradoxically, apoptosis of acinar cells has shown to be somewhat protective in AP, which may be due to a reduction in DAMP release in response to apoptosis compared with necrotic cell death.^102–104^ As necrosis and/or excessive autophagy of acinar cells occurs, contents within these cells are released into the environment, including several DAMPs: IL-33, high mobility group box 1, and ATP.^105^ All of these molecules are known activators of the innate immune system and have been proposed to be the critical molecules initiating disease in AP.^105^

Interleukin 33, typically stored in the nucleus of the cell, is passively released from cells upon cell necrosis and/or during tissue damage and acts as an “alarm” for the immune system.^106^ Duct ligation-induced AP in both mice and rats has been shown to induce elevated levels of IL-33 in the pancreas.^107^ In addition, intraperitoneal injections of IL-33 alone induced neutrophil and macrophage infiltration coupled with perivascular edema.^107^ Chen et al^108^ found that pancreatic ductal administration of an adenoviral vector that activates NF-κB is sufficient to induce AP and is reduced via coadministration of an NF-κB inhibitor. It is likely that the effect of the activation state of the NF-κB pathway is immune cell specific. More specifically, the pathways activated in neutrophils are particularly interesting as the depletion of these cells in a taurocholate-induced mouse model of AP reduces pancreatic injury and inflammation.^109^ In addition, high mobility group box 1 is elevated in pancreatic tissue throughout AP and has been shown to contribute to AP progression in a toll-like receptor 4 and NF-κB–dependent manner.^110^ Extracellular ATP has been found to be increased in the circulation of mice regardless of the method of AP induction, and this ATP was found to stimulate neutrophil migration and activation.^111^ The increase in released ATP may be due to the dysfunctional Ca^2+^ signaling observed in acinar cells and is known to contribute to mitochondrial dysfunction and improper ATP production.^112^ Taken together, DAMPs released in response to acinar cell damage provide a potent proinflammatory environment in the tissue to perpetuate disease progression.

In addition to direct stimulation of neutrophils and other inflammatory cells, DAMPs can activate the complement cascade, a critical component of innate humoral immunity. This complement system has been extensively studied in the context of AP because of its known role in mediating local and systemic inflammation.^113^ Recently, the critical complement component 3 (C3) was identified as a key regulator of neutrophil recruitment and activation in a taurocholate-induced mouse model of AP.^114^ In addition, C5 has been determined to stimulate pancreatic stellate cells in 2 mouse models of chronic pancreatitis, indicating that this system may activate both inflammatory cells and pancreatic stromal cells in the disease.^115^ In humans, elevated levels of C3a and sC5b-9 are observed early in AP and act as a strong predictor of pancreatic necrosis with a sensitivity of 0.93 and sensitivity of 0.88.^116^ Interestingly, C5a has also been found to exert anti-inflammatory properties in the context of AP^117^ and sepsis^118^ suggesting that the complement cascade is a complex communication network, which must be further studied to properly target in AP patients.

Another cytokine implicated in AP is IL-8, which is predictive of severe AP in patients with similar sensitivity and specificity as IL-6.^119–121^ However, mechanistic studies involving IL-8 in AP are lacking, and only a weak association between an IL-8 polymorphism and AP has been identified.^122^ The interferon-γ–inducing factor IL-18 is another proinflammatory cytokine, which has been shown to activate the NF-κB pathway.^123^ Circulating IL-18 is significantly upregulated in AP patients as early as day 1 and as late as day 5 compared with healthy controls.^124^ In addition, IL-18 is positively correlated with several other inflammatory markers found in circulation in AP patients (CRP, IL-6, IL-8).^124–126^

In conclusion, the inflammatory response in AP is complex, with several cascades and cytokines mediating cellular-specific outcomes to maintain a proinflammatory environment in the tissue, which becomes systemic. Furthermore, whether DAMPs precede the acute phase response in AP is unclear. Therefore, therapeutic targets that reduce DAMP and acute phase protein production or signaling may provide the most significant potential.

## POTENTIAL THERAPEUTIC STRATEGIES

**FIGURE 1:**
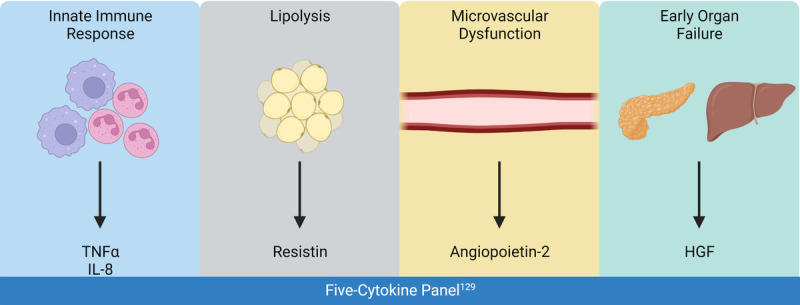
The 4 arms of the 5-cytokine panel. The 5-cytokine panel that accurately predicts patient outcomes in severe acute pancreatitis considers several aspects of the response driving persistent organ failure.^128^ Figure created with BioRender.com.

Despite the development of minimally invasive drainage/debridement techniques for pancreatic necrosis in SAP, there have been little to no improvements in patient outcomes.^127^ Recently, a 5-cytokine panel including angiopoietin 2 (Ang-2), hepatocyte growth factor (HGF), IL-8, resistin, and TNF-receptor superfamily IA has been reported to accurately predict persistent organ failure (POF) in AP with a 10-fold cross-validated accuracy of 0.89.^128^ Each cytokine was carefully chosen for this panel because of their specific roles in the POF cascade including activation of the innate immune system (TNF-α, IL-8), lipolysis of peripancreatic/intrapancreatic fat (resistin), microvascular dysfunction (Ang-2), and early organ injury (HGF) (Fig. 1).^128,129^ In addition, these measurements can be taken within 24 hours from the onset of the symptoms to predict POF in AP, which may significantly improve future clinical trials that require early identification of SAP patients.^128^ These findings suggest therapeutic strategies and clinical interventions focused on circulating cytokines may significantly benefit SAP patients as these molecules are targetable and upregulated early in disease. Future clinical trials may consider targeting several of these 5 cytokines to inhibit disease progression and POF.

### Interleukin-10 Master Regulator

Interleukin 10 has been extensively studied in the context of several inflammatory disorders and has been identified as a critical regulator of tissue homeostasis by restricting excessive inflammatory responses.^130^ Indeed, several studies have determined that IL-10 is produced by nearly all subsets of leukocytes, including macrophages,^131^ T-cells,^132^ and neutrophils^133^ to signal in an autocrine/paracrine manner to exert anti-inflammatory effects on these cells. More specifically, IL-10 has been determined to reduce cytokine production of several of the inflammatory cells found in AP, providing an attractive therapeutic target.^134^ Multiple animal studies have identified that exogenous administration of IL-10 reduces chemically induced SAP.^135,136^ However, a clinical trial performed in 2001 identified no benefit from 8 μg/kg of IL-10 for endoscopic retrograde cholangiopancreatography–induced pancreatitis indicating that alternative cytokine regulators must be identified and targeted to prevent POF in SAP patients.^137^ Interestingly, a recent study determined that administration of pirfenidone increases IL-10 production in a mouse model of AP, which may provide an alternative method for increasing circulating IL-10 in SAP patients and should be further explored in humans.^138^

### Alternative Cytokine Targets

Activin A, a member of the transforming growth factor β superfamily, is a cytokine with critical roles in cell differentiation, cancer, secretion of other cytokines, and inflammation.^139–142^ The cytokine cascade observed in severe AP patients with SIRS is similar to sepsis, a critical illness where serum activin A is a predictor of sepsis severity in early disease.^143^ In addition, activin A is released in response to LPS downstream of the DAMP receptor toll-like receptor 4, indicating that activin A may be a critical component of the acute phase response.^144^ Activin A has been shown to induce IL-1β, IL-6, and TNF-α production in peripheral blood mononuclear cells from human donors.^145^ In addition, recent evidence indicates activin A signals in an autocrine manner to drive IL-6 production in cancer cells, further supporting the notion that activin A drives IL-6 production and the initial inflammatory events leading to the acute phase response.^146^

Activin A seems to interact with several of the cytokines included in the 5-cytokine panel, which provides an attractive target for potential AP therapeutics. Administration of TNF-α to isolated human neutrophils induces activin A secretion, indicating that neutrophils may also provide a significant source of activin A in an inflammatory setting.^147^ Inhibition of activin A signaling reduced TNF-α secretion and inflammatory infiltrate in a mouse model of malaria infection, further supporting to the proinflammatory role of activin A.^147^ These data suggest a potential positive feedback loop in which TNF-α enhances activin A production and vice versa to enhance the inflammatory response. Moreover, IL-8 production is induced in human endometrial stromal cells when activin A is administered, further confirming the proinflammatory role of the molecule.^148^ Activin A has been shown to activate several pathways but has been classically identified to activate the SMAD pathway, where SMAD4 translocation into the nucleus results in transcriptomic changes.^149^ Inhibition of SMAD4 has recently been shown to increase transcription for the gene encoding Ang-2 providing evidence that activin A may inhibit Ang-2 production and reduce vascularization to the injured pancreas in AP.^150^ There is little to no evidence explaining the potential relationship of activin A to HGF or resistin, and further research is required to elucidate what interactions may exist.

These data suggest that activin A promotes a significant upregulation in several cytokines, which increase in the circulation of AP patients. In addition, the role of activin A in enhancing TNF-α and IL-8 production while reducing the effects of Ang-2 suggests that this molecule may promote inflammation while reducing pancreatic vascularity for repair.

Pancreatic acinar cells have been shown to produce significant amounts of activin A in a mouse model of cerulein-induced AP with little production under homeostatic conditions, suggesting a specific role for activin A in response to pancreatic insult.^151^ Clinical data indicate that AP patients' circulating activin A levels are increased compared with controls, independent of body mass index.^152^ This study also found that levels of circulating activin A raised in a stepwise fashion relative to disease severity, with the highest amount of activin A found in severe AP patients.^152^ Importantly, high levels of activin A at admission were predictive of a more extended hospital stay when compared with low and intermediate levels.^152^ Activin A seems to play a critical role in the mechanism driving disease severity in in vivo models of AP. *Ob/ob* mice injected with cerulein develop severe AP, which is reduced in anti–activin A–treated mice that also display an increased survival rate in this model when compared with vehicle-treated controls.^152^ Anti–activin A intervention has been shown to reduce pancreatic inflammation and tissue damage.^153^ Pancreatic stellate cells are also a source of activin A production in the inflamed pancreas.^153^ Upon stimulation, neutrophils have also been shown to produce large quantities of activin A, which can signal in an autocrine/paracrine fashion.^147^ Previous research has identified that when neutrophils are depleted in mice, AP development is diminished with reductions in the inflammatory infiltrate and tissue damage demonstrating an attractive immune cell target for anti-AP therapeutics.^154^

These data suggest that anti–activin A intervention will improve SAP patient outcomes through several distinct anti-inflammatory pathways. In addition, anti–activin A therapy is well tolerated in humans providing excellent potential for future anti–activin A clinical trials.^155^

## CONCLUSIONS

**FIGURE 2:**
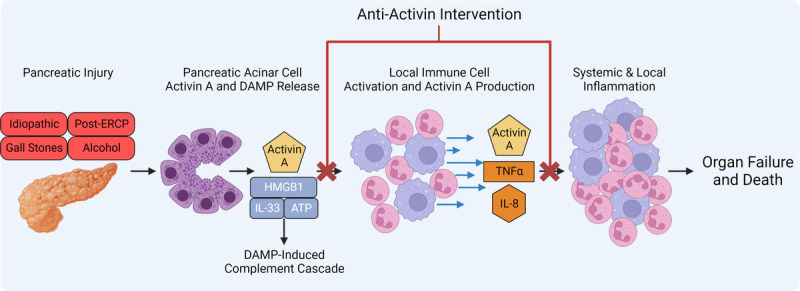
Early pancreatic injury may result in pancreatic acinar cells to release activin A and DAMPs into the microenvironment. The DAMPs can activate the complement cascade while simultaneously promoting inflammation in the tissue. Activin A signaling along neutrophils and macrophages can further stimulate activation and recruitment of these cell types to the pancreas where they can further produce, release, and respond to activin A. This leads to a proinflammatory cytokine cascade within the tissue that becomes systemic leading to organ failure and death. Antiactivin therapeutics have significant potential in both early and late stages of AP to reduce activin A signaling on immune cells to reduce inflammatory processes. This molecule may be driving both the initial response of immune cell recruitment to the tissue and the persistent inflammatory response locally and systemically. Figure created with BioRender.com.

Acute pancreatitis remains a major clinical challenge because of the lack of therapeutic options to prevent disease progression. The significant knowledge gap surrounding AP mechanisms is due to the complex nature of the disease and the shortage of well-designed clinical trials.^3^ The application of a 5-cytokine panel seems to have the most significant potential in predicting patient outcomes (Fig. 1), which should be leveraged for future clinical trials.^128^ The success of this panel suggests that a molecular approach designed to inhibit the production of several inflammatory mediators may provide tremendous success in the clinic. Activin A seems to promote the production and secretion of several critical inflammatory cytokines associated with the cytokine cascade observed in AP.^152,153^ Given that activin A regulates several cytokines of interest in AP and anti–activin A therapeutics are well tolerated in humans, therefore activin A is an attractive target for clinical trials (Fig. 2).^155^ Future research should identify the mechanism by which activin A promotes disease progression. Alternatively, targeting Ca^2+^ signaling may provide significant therapeutic potential given the preliminary success of Auxora.^67^ More human studies must be completed to determine the safety and efficacy of targeting Ca^2+^ signaling via Orai1 inhibition.
